# Modeling Na^+^- and Ca^2+^-dependent mechanisms of rhythmic bursting in excitatory neural networks

**DOI:** 10.1186/1471-2202-13-S1-P38

**Published:** 2012-07-16

**Authors:** Ilya A Rybak, Patrick E Jasinski, Yaroslav I Molkov, Natalia A Shevtsova, Jeffrey C Smith

**Affiliations:** 1 Department of Neurobiology and Anatomy, Drexel University College of Medicine, Philadelphia, PA 19129, USA; 2 Department of Mathematical Sciences, Indiana University – Purdue University Indianapolis, IN 46202, USA; 3 Cellular and Systems Neurobiology Section, National Institute of Neurological Disorders and Stroke, National Institutes of Health, Bethesda, MD 20892, USA

## 

The mechanisms generating neural oscillations in the mammalian brainstem, particularly in the pre-Bötzinger complex (pre-BötC) involved in control of respiration, and the spinal cord (e.g. circuits controlling locomotion) that persist after blockade of synaptic inhibition, remain poorly understood. Experimental studies in medullary slices from neonatal rodents containing the pre-BötC identified two mechanisms that could potentially contribute to generation of rhythmic bursting in the pre-BötC: one based on the persistent sodium current (*I_NaP_*) [1,2], and the other involving the voltage-gated calcium (*I_Ca_*) [3] and/or the calcium-activated nonspecific cation current (*I_CAN_*), activated by intracellular Ca^2+^ accumulated from extra- and/or intracellular sources [4]. However, the involvement and relative roles of these mechanisms in rhythmic bursting are still under debate.

**Figure 1:**
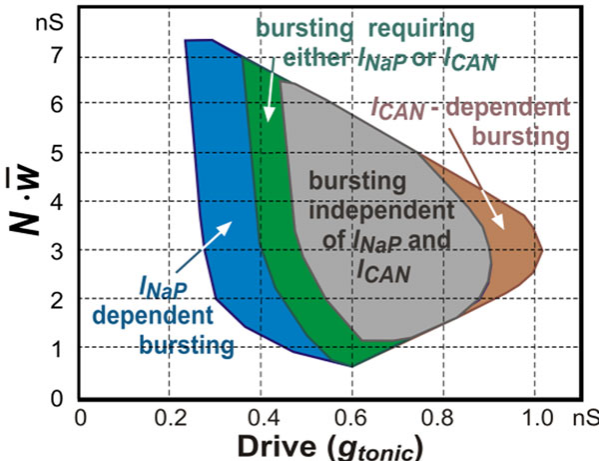
A map of bursting activities derived from multiple simulations of the excitatory neuron population and presented in a 2D parameter space (*g_tonic_*, ), where *g_tonic_* is the external excitatory drive to all neurons, *N* is the number of neurons in the population, and  is the average weight of mutual excitatory synaptic interactions within the population. Bursting activities involving different mechanisms are distinguished by color. The area for *I_NaP_*-dependent population bursting is shown blue; the area in which population bursting may be based on either *I_NaP_* or *I_CAN_* is shown green; the area in which population bursting may exist without both of these currents is shown gray; an additional area (brown) represents an unstable *I_CAN_*-dependent bursting.

In this theoretical/modeling study we investigated Na^+^- and Ca^2+^-dependent bursting generated in single cells and in a heterogeneous population of synaptically interconnected excitatory neurons with *I_NaP_*, and *I_Ca_* randomly distributed within the population. We analyzed the possible roles of network connections, ionotropic and metabotropic synaptic mechanisms, intracellular Ca^2+^ release, and the Na^+^/K^+^ pump in rhythmic bursting activity generated under different conditions. We show that the heterogeneous population of excitatory neurons can operate in different oscillatory regimes with bursting dependent on *I_NaP_* and/or *I_CAN_*, or independent of both (Fig. 1). The oscillatory regime and operating bursting mechanism may depend on neuronal excitability, synaptic interactions and relative expression of particular ionic currents.

The existence of multiple oscillatory regimes and their state-dependency may provide explanations for different rhythmic activities observed in the brainstem and spinal cord under different experimental conditions.
